# Gut and Vaginal Microbiomes in PCOS: Implications for Women’s Health

**DOI:** 10.3389/fendo.2022.808508

**Published:** 2022-02-23

**Authors:** Yuanyuan Gu, Guannan Zhou, Fangyue Zhou, Yao Li, Qiongwei Wu, Hongyu He, Yi Zhang, Chengbin Ma, Jingxin Ding, Keqin Hua

**Affiliations:** ^1^Changning Maternity and Infant Health Hospital, East China Normal University, Shanghai, China; ^2^Department of Gynecology, The Obstetrics and Gynecology Hospital of Fudan University, Shanghai, China; ^3^Department of Gynecology, Shanghai Key Laboratory of Female Reproductive Endocrine Related Diseases, Shanghai, China; ^4^The International Peace Maternity and Child Health Hospital, School of Medicine, Shanghai Jiao Tong University, Shanghai, China; ^5^Department of Urology, Gongli Hospital of Shanghai Pudong New Area, Shanghai, China; ^6^Department of Intensive Care Unit, Zhongshan Hospital, Fudan University, Shanghai, China

**Keywords:** PCOS (polycystic ovarian syndrome), gut microbiomes, vaginal microbiomes, mechanism, therapeutics

## Abstract

PCOS is defined as a kind of endocrine and metabolic disorder which affects females at reproductive ages, is becoming much more common, nowadays. Microbiomes are known as microorganisms that inhabit the body to play a vital role in human health. In recent years, several basic and clinical studies have tried to investigate the correlation between the reproductive health/disorder and microbiomes (gut microbiomes and vaginal microbiomes). However, the mechanism is still unclear. In this review, we reviewed the relationship between PCOS and microbiomes, including gut/vaginal microbiomes compositions in PCOS, mechanism of microbiomes and PCOS, and then collectively focused on the recent findings on the influence of microbiomes on the novel insight regarding the therapeutic strategies for PCOS in the future clinical practice.

## Introduction

Polycystic ovary syndrome (PCOS) (1, 2) is widely defined as a kind of endocrine and metabolic disorder with a combination of signs and symptoms of androgen excess (hirsutism and/or hyperandrogenemia) and ovarian dysfunction (oligo-ovulation and/or polycystic ovarian morphology (PCOM)) (3). It was reported that the prevalence of PCOS in premenopausal women is up to almost 20% as the current, more inclusive definitions (4–6), which suggesting PCOS the most common endocrine and metabolic disorder in women at reproductive age.

As the increasing studies aiming at the microbiota, it is widely believed that microbiota has evolved together with the hosts and is becoming an integral part of the human body (7). It is well acknowledged that the microbiota is responsible for more than 95% of the genetic activity of the organism (8). The microbiota were called as “second genome” for the human body, indicating that microbiota exerts vital function in human health (9). Various kinds of microbiota are acknowledged to play roles in influencing physiology balance (10), metabolism process (11), nutrition production (12), and immune mediation (13) under physiological conditions. The complex and delicate balance between the microbiota and the host maintain the health of human. Despite there are various evidences to demonstrate the strong associations between human health and diverse types of microbiota including gut microbiota and vaginal microbiota, the molecular mechanisms are still unclear. Thus, the clear descriptive function from diverse microbiota is still unclear worth further exploring.

In this review, we summarized the existing research on microbiota in PCOS disorder. We aimed to illustrate the relationship between the various microbiota (referring to both the gut microbiota and the vaginal microbiota) and PCOS. Moreover, we summarized the mechanisms of microbiota participate in the development of PCOS and the applications of microbiota in treating PCOS, which might be potential insights for the intervention of PCOS and other related endocrine and metabolic disorders.

## Materials and Methods

**Figure 1:**
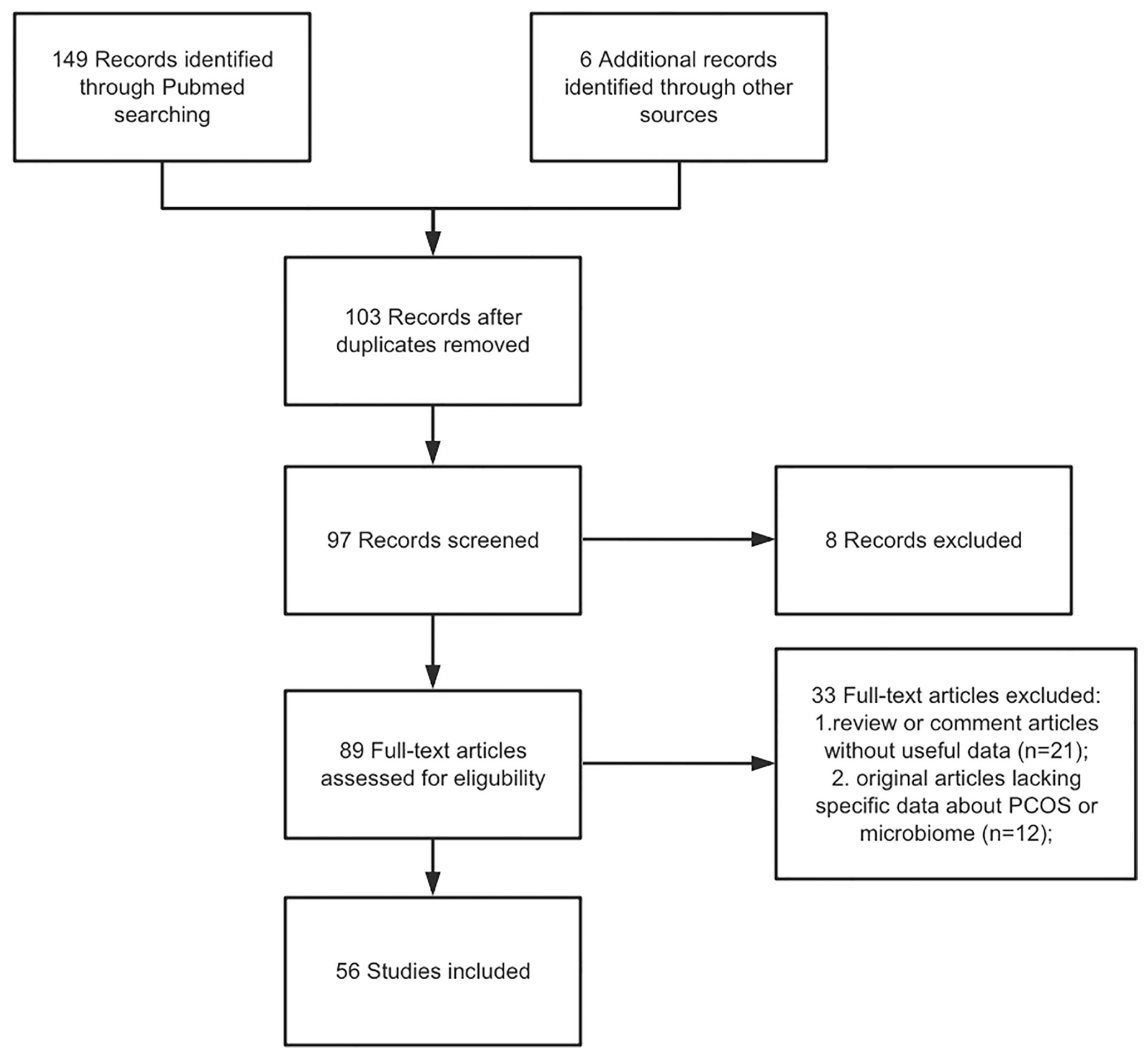
Flow diagram of the searching strategy.

A literature enrollment on the PUBMED database for articles in English and published from inception to November 2021 was performed. We used the Medical Subject Headings (MeSH) terms to screen the target studies: “PCOS”, “microbiome”, “molecular mechanism”, “gut”, “vaginal”, “insulin resistance”, and “therapeutic strategies”. Non-English articles, or abstract only, and studies including information that overlapped other publications were excluded. In our search, only articles concerning PCOS were included. The selection criteria included original articles as well as review articles regarding the PCOS and microbiome. Articles that met the inclusion criteria were carefully read, and, if appropriate, further articles cited in the references were enrolled. **Figure 1** demonstrated the flow chart of article selection.

## PCOS

Polycystic ovary syndrome (the common abbreviation is “PCOS”) is widely acknowledged as an endocrine disorder that affects almost 10% of reproductive age women. PCOS is characterized by hyperandrogenism (4), ovarian dysfunction, and metabolic syndrome. The main symptoms including hirsutism, irregular menstrual periods, and ovarian cysts. As the deepening of the studies, PCOS is also regraded as a kind of metabolic disease, with the symptoms including increased triglycerides, low-density lipoprotein cholesterol and insulin resistance indices (14). Since the amount of females disturbed by PCOS increases and the mechanism of PCOS is still unclear, there is an increasing studies conducting relevant experiments. Many studies have revealed the relevance of the relationship between the alteration of pathogenic factors (15) (including lifestyle, obesity, genetic factors and so on) and PCOS. However, few studies have explored the relationship between microbes and PCOS, especially the vaginal microbes and PCOS.

## The Microbiota

Recent years, increasing studies focus on the microbiota and human healthy (16). The microbiota community is now well acknowledged as a complex ecosystem of microorganisms including bacteria, viruses, protozoa and fungi. The microbiota communities (17) exist in almost all the districts of the human body including gastro-enteric track (gut microbiota), skin (skin flora), mouth (oral flora), respiratory system (respiratory tract microbiota), and the vagina (vaginal microbiota). Each microbiota community plays vital role in regulating the homeostasis *via* different kind of pathways in numerous systems.

### The Gut Microbiota

It was reported (18) that the human gut microbiome consisted of about 10^13^ and 10^14^ micro-organisms. Among these micro-organisms, more than 1000 different kinds of species and more than 7000 different kinds of strains were classified and named, including bacteria, viruses, protozoa, archaea, and fungi (19). Even though the original and establishment process of gut microbiota in early life is still an undefined issue, it is widely acknowledged that microbiota begins to develop immediately after birth and is influenced by many factors such as age, diet, lifestyle and so on. The human gut microbiota is mainly composed of five bacterial phyla: Firmicutes phylum, Bacteroidetes phylum, Proteobacteria phylum, Actinobacteria phylum and Verrucomicrobia. Of all the five bacterial phyla, Firmucutes and Bacteriodetes account for almost 90% and Actinobacteria and Proteobacterium account for 10%, while Verrucomicrobia accounts for the smallest proportion.

Numerous of gut microbiota are reported to regulate the physiology balance in numerous ways including in metabolic protective, structural integrity (20) and histological homeostasis. On the contrary, disruption of the composition of intestinal microbiota (a decrease or increase in the ratio of beneficial or harmful bacteria) is associated with many diseases or disorders. The gut microbiota plays vital roles in the metabolic process include the production of vitamins, short-chain free fatty acids and conjugated linoleic acid, as well as the biotransformation of bile acids, ammonia synthesis, and detoxification. The gut microbiota is proved to be involved in the production of acetate (21), butyrate, and propionate (22), which are main short-chain fatty acids with effect of anti-inflammatory, anticarcinogenic, and immunomodulatory. Also, the gut microbiota is proved to be involved in the metabolism of Butyrate and Propionate, which mediates the energy metabolism by regulating the gluconeogenesis process and cholesterol metabolism. The gut microbiota also has been proved to be associated with the regulation and modulation of the immune system. Some studies reported that the gut bacteria might participate in the development of T cells (13), and Th-17 cells in the development of immune system. In views of the effect of gut microbiota on the structural functions, some studies reported that gut microbiota is involved in maintaining epithelial integrity through regulating tight junction expression. While the balance of gut microbiota is in a dynamic status, some basic effects on immunological functions, metabolic process, structural integrity of the human body is associated with the gut microbiota. A better understanding of the functioning of gut microbiota would undoubtedly led to some very exciting developments in therapeutics to improved health.

### The Vaginal Microbiota

The vaginal microbiota has drawn numerous attention since it was regarded as being shaped over the years by co-evolutionary processes and playing vital role in female health. It has been found that in the majority of vaginal microbiota is dominated by the Lactobacillus bacterial species (23), which prevent the colonization of harmful bacteria *via* producing hydrogen peroxide (24) in the microenvironment. Also, a series of diversified strictly and facultative anaerobic microbes were found in healthy women, suggesting that there are multiple microbiomes instead of a single microbiome in the human vagina. The vaginal microbiota is grouped as five communities: i) the CST I [dominated by Lactobacillus crispatus (L. crispatus)]; ii) CST II (dominated by L. gasseri); iii) CST III (dominated by L. iners); v) CST V (dominated by L. jensenli) and iv) CST IV (lacks Lactobacillus sp. and contains huge amounts of strict anaerobic bacteria like Megasphera, Prevotella, Gardenella and Sneathia). The vagina in normal situations is acidic with low pH value because of the presence of hydrogen peroxide and lactic acid secreted by Lactobacillus sp. Vaginal secretions contain numerous microorganisms and the host provides them nutrients for their growth and development. Disruptions in vaginal association with the microbiomes lead to the change in the vaginal environment, which enhanced the risk of female related disorders or female related diseases.

## Microbiota Composition in PCOS

Since microbiota communities are in dynamic equilibrium in healthy women, the unbalanced microbiota composition is regarded as be associated with the PCOS women. It has been widely demonstrated by many researchers that microbiota composition changes and dysbiosis (25) occurs in PCOS animal models and women with PCOS.

### Vaginal Microbiome in PCOS Patients

It was reported that there are large differences in vaginal microbiome between pre-pubertal women and postmenopausal women (26). It is mainly result from the lower genital tract microbiome would be affected by the age, sex hormones level, living habits and so on (25, 27). Among these factors, irregular menstruation and abnormal hormone levels are acknowledged as the two main reasons leading to the alteration of vaginal microbiome in PCOS women. Normal menstruation with regular changes in estrogen and progesterone would drive the physiological changes in the epidermal cells in reproductive tract and maintain the balanced microenvironment (including the balanced microbiota community). On the contrary, the irregular menstruation in PCOS women would lead to the alternation about the composition of lower genital tract microbiomes. Some studies investigated the lower genital tract microbiome composition of PCOS women and healthy women. The results derived from 194 microbial samples which analyzed by the 16S rRNA gene sequencing (28) indicate that there is a significant difference of taxa abundance between PCOS and healthy women in both vaginal microbiomes and cervical canal microbiomes. In PCOS women, the results witnessed a significantly decreased composition of Lactobacillus (**Table 1**). On the other hand, some other microbiomes such as Gardnerella vaginalis (29), Chlamydia trachomatis and Prevotella increased at the same time. Moreover, these increased microbiomes are regarded as potential pathogenic taxa in the vagina and cervical canal.

**Table 1:** Summary of composition changes of microbiomes in PCOS.

Microbiomes composition	Outcomes
**Gut Microbiomes**	
Decrease of α diversity	Girls with PCOS had decreased α-diversity compared to non-PCOS.
Decrease of β diversity	The β diversity of PCOS was significantly decreased.
**Vaginal Microbiomes**	
Decreased of Lactobacillus	In PCOS women, Lactobacillus decreased significantly.
Increased of Chlamydia trachomatis	In PCOS women, Lactobacillus decreased significantly.
Increased of Prevotella	2 2In PCOS women, Lactobacillus decreased significantly.

The results are in line with many previous related studies (27, 30), which also suggested that the decrease of Lactobacillus spp. is associated with infertility, abortion, recurrent implantation failure, and some other adverse pregnancy outcomes. Moreover, some studies have reported that Gardnerella and Prevotella species (31) are related to bacterial vaginosis (BV), which would decrease the probability on the procedure of embryo implantation and even the growth of the fetus. These alternations in the composition of microbiota might be linked to the fact that PCOS women are often disturbed by infertility, abortion, and several other adverse reproductive outcomes.

### Gut Microbiome in PCOS Patients

Different studies have found that some microbiota changes in PCOS women at phylum, family, and genus level, respectively. A pilot study reported (32) the abundance of Tenericutes phylum, ML615J-28 and S24-7 decreased in PCOS women. Studies also found the decreased abundance of Akkermensia and Ruminococcaceae (33), while the increased level of Bacteroides and Escherichia/Shigella in women with PCOS. Interestingly, studies found that unbalanced gut microbiota in PCOS women is similar with that of the obese non-PCOS women (34), which indicated some unclear relationship between the microbiota composition in obesity and in PCOS.

When focus on the phylum level, it was reported that the proportion of Actinobacteria is larger in PCOS women, and the proportion of Bacteroidetes is smaller in PCOS women when compared with the healthy controls. Some other studies reported that they found the phylogenetic diversity of the phylum Bacteroidetes decreased (25) significantly when compared with healthy controls (**Table 1**). Further studies found that transplantation of fecal microbiota from women with PCOS would lead to the elevated proportion of disrupted ovarian functions, insulin resistance and infertility [15]. Also, some studies reported that Bacteroides were enriched in the PCOS women (35). Zhang et al. found that the larger proportion of Bacteroides in PCOS women by analyzing the stool microbiome. Bacteroides, as a kind of pro-inflammatory bacteria, is reported be associated with promoting the insulin resistance (36), hormonal disturbance, and inflammation in PCOS women.

Given this, we speculated that microbiota might be involved in the pathogenesis of PCOS by promoting insulin resistance, driving the fluctuation of sex hormones, regulating the immune balance and other pathological mechanisms.

## Relation of Microbiota and PCOS

### Microbiome and Sexual Hormones in PCOS

**Figure 2:**
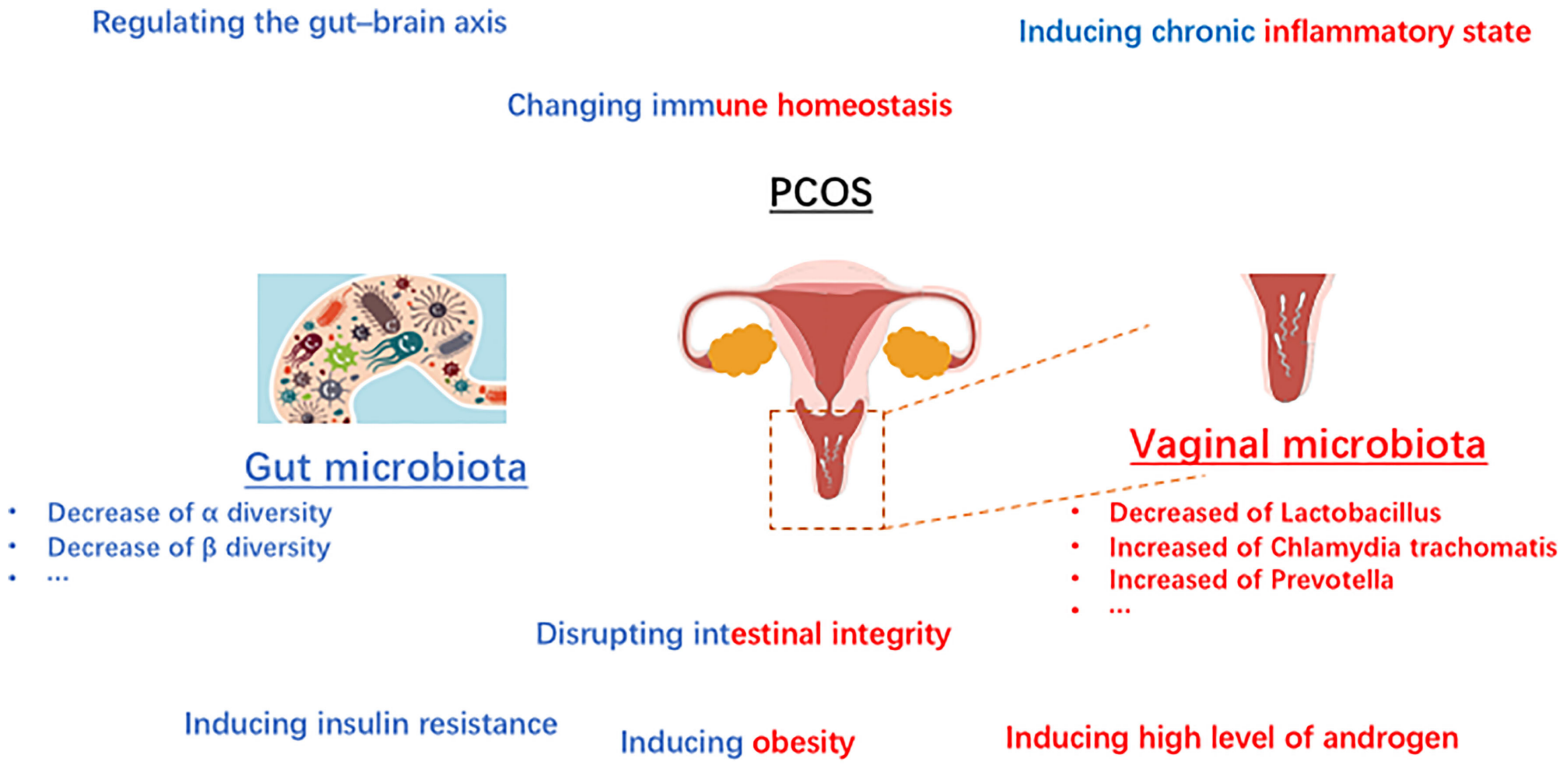
Schematic diagram showing the composition changes and mechanisms microbiota in PCOS.

Recent studies have shown that sex hormones (25) influence the composition of the microbiome, including gut microbiome and vaginal microbiome. In the PCOS situation, sexual hormones might also play a role in regulation of the microbiome. Unbalanced level of hormones may be associated with the “out-of-balance” of microbiome in PCOS. Gut microbiome, as well as vaginal microbiota play vital roles in regulating sexual hormones. It was widely acknowledged that estrogens are metabolized through the liver and then excreted *via* urine (37). The glucuronic acid allows estrogens to remain in the body and exert their effects, which plays a vital role in the process. It was reported that β-glucuronidases and β-glucuronides produced by the genera Bifidobacterium (38), Clostridium, and Lactobacillus, and play roles in the de-conjugation/conjugation of estrogens (39). The bacteria derived from gut microbiome involve in the metabolism of hormones *via* producing relative enzymes and thus regulate the circulation of sexual hormones in women. Increasing studies have reported that estrogens play a vital in establishing the balanced microbial community structure in women (40). What is widely acknowledged that estrogens play a key role in increasing the glycogen production in vaginal epithelial cells, and subsequently promoting the growth of Lactobacillus. Even though some studies reported that the high starch diets are responsible for the developing the dominant status of Lactobacillus (41), estrogen is still regarded as the core factor in the process. What’s more, microbiome also regulate the sexual hormones in PCOS. It was reported that the high level of androgen in women with PCOS is associated with metabolic dysregulation (42). Since the sex hormone is affected by microbiome, it is reasonable to speculate that microbial imbalance is responsible for PCOS. Several studies reported that PCOS was linked to the abnormal fluctuations of the gut microbiota composition (43). These fluctuations could be briefly summarized as the abnormal changes of beta diversity, and the decline of alpha diversity including not only the species richness but also the phylogenetic diversity. In addition, several studies have reported that lower level of alpha diversity of the gut microbiome might be related to the obesity (44). It is worth further investigating how the abnormal microbiota composition regulated the metabolic process in women with PCOS. What’s more, the interaction between sex hormone, the vaginal as well as the gut microbiomes is a multi-step process. The gut microbiomes regulate the level of estrogens, and the estrogens moderate the vaginal microbiome. Thus, the abnormal gut microbiomes and/or vaginal microbiomes might affect each other in PCOS, including regulating the composition of microbiome and moderating the changes of hormones. The immune homeostasis is beneficial to establish the healthy microenvironment in female (**Figure 2**).

### Microbiota and Immune Homeostasis in PCOS

It is worth noting that numerous kinds of microbiota in human body participant in the homeostasis modification, particular in regulating the immune homeostasis (45, 46). On the one hand, as the increasing studies focusing on the mechanism of gut microbiota in healthy, the interactions between the gut microbiota and immune-related influence are becoming better known. The researchers reported that the intestinal immune system is mainly shaped by the gut microbiota (47). Among the intestinal immune system, myeloid cells are regarded as the first immune responders and the effects of gut microbiota on intestinal macrophages has been implicated by multiple studies (48–50). Studies reported that in germfree animals, an array of intestinal immune defects including impaired development of gut-associated lymphoid tissues, gut-associated Th17 cells, lower numbers of IgA-producing B cells and intraepithelial CD8+ T cells were widely observed (51). On the other hand, there is an increasing body of evidence that the disordered immune system which affected by the vaginal microbiota is associated with the diseases in vaginal districts (**Figure 2**). Considering the anatomical complexity of the female genital tract which near to the intestine, the vaginal bacteria community might be influenced by the gut microbiota (45, 52). In addition, that the vaginal microbiota also be regulated by the numerous chemical changes in microenvironment as well as the periodically hormonal fluctuations. The influence of the vaginal microbiota upon the female immune system is mainly to prevent external pathogens infections as well as to maintain an immuno-tolerant environment. Once the dominance of Lactobacillus is disrupted, the immune homeostasis changed including producing pro-inflammatory cytokines, abnormal the immune cells recruitment and so on.

### Gut Microbiome and Insulin-Resistance in PCOS

Insulin resistance, is widely defined as an endocrine disease (53, 54). People with insulin resistance could not increase glucose uptake and utilization as a normal population because the inability of a quantity of insulin (exogenous or endogenous). Recently years, insulin resistance is widely acknowledged a kind of metabolic disorder related to the gut microbiome not only in animals but also in humans. It has been shown that the level of serum branched chain amino acids in insulin-resistant individuals is significantly elevated, which is associated with the gut microbiome in PCOS (43). The results indicate that *Prevotella copri* and *Bacteroides vulgatus* are two main species mediating the biosynthesis of BCAAs and promoting the insulin resistance (36). Furthermore, insulin resistance could induce hyperinsulinemia, and then drive the excessive androgen production by the ovaries (**Table 2**). In addition, hyperinsulinemia derived by insulin resistance could increase the level of testosterone by reducing sex hormone binding globulin (55). In addition to the above-mentioned mechanisms, short-chain fatty acid (SCFA) is reported that be involved in the pathogenesis of PCOS *via* promoting insulin-resistance (56). SCFAs (including acetate, propionate, and butyrate) have been verified to participant in the metabolism process, and exert the functions including inhibiting inflammatory, and regulating immune balance (57). Some studies reported that the decreased level of SCFA might be related to the development of insulin-resistant (58), which plays an important role in the development of PCOS (**Figure 2**). Besides, it has been suggested that bile acid is related to microbiome in PCOS women (59). Gut microbiota could regulate bile acid metabolism including in synthesis, metabolism, and reabsorption of bile acids, through vitamin D receptor and G protein-coupled receptor (60). Some studies found that gut microbiota-bile acid-interleukin-22 axis plays an important role in the development of PCOS (43). In brief, the levels of bile acid (including glycodeoxycholic acid and tauroursodeoxycholic acid) as well as the level of interleukin-22 was also decreased in the PCOS women.

**Table 2:** Summary of the mechanism of microbiomes changes and PCOS.

Mechanism	Microbiomes changes
Inducing high level of androgen	Decreased alpha diversity
Inducing obesity	Decreased alpha diversity
Changing immune homeostasis	Disrupted Lactobacillus
Inducing insulin resistance	Increased Prevotella copri
Inducing insulin resistance	Increased Bacteroides vulgatus
Regulating the gut–brain axis	Unbalanced gut microbiota community
Inducing chronic inflammatory state	Unbalanced gut microbiota community
Disrupting intestinal integrity	Unbalanced gut microbiota community promoting release of pro-inflammatory factors

### Gut-Brain Axis in PCOS

Increasing studies witnessed the development of research of the brain disorders [including anxiety, depression, and Alzheimer’s disease (61)], which are verified be associated with the gut microbiome (62). In recent years, many studies demonstrate the correlation between the gut and the brain, particular depict the vital role of the gut-brain axis (63), which exert important roles in communication between the gut microbiome and the brain. Studies reported that females are more likely been disturbed by the anxiety disorders not only the frequency of occurrence but also the degree of severity, when compared with the males (64).

Considering the communications for information between the central nervous system and the gastrointestinal system, the microbiota is believed to play roles in affecting the brain-gut axis in various ways. On the one hand, gut microbiota could stimulate the vagal pathways to send signals to communicate with the brain, directly. On the other hand, the microbiota could send the messages to the brain *via* releasing various complex substances (65) and influence the synapses formation (66). What’s more, gut microbiota could regulate the gut–brain axis through mediating the immune pathways. In addition, unbalanced gut microbiota community could participant in forming abnormal hormone changes by promoting anxiety disorders.

### Other Mechanism in Microbiota Leading to PCOS

#### Chronic Inflammatory State

The chronic inflammatory results from the unbalanced gut microbiota might be associated to the development of PCOS. Several studies have reported that the gut microbiota disorders can accelerate the process of producing LPS and promote the production of TNF-α, IL-6 and so on (67), which are widely recognized as inflammatory factors and could induce the further insulin resistance. When considering the molecular mechanism in these process, it was demonstrated that the chronic inflammation is related to the hyper-androgens status and also play vital role in obesity development, which are responsible for the abnormal development of normal follicles (68).

#### Intestinal Permeability

One of some other mechanisms of gut microbiota in the development of PCOS is that the unbalanced gut microbiota community mediate the disrupting process of the intestinal mucosal integrity. The unbalanced microbiota produce more pro-inflammatory cytokines, and the increased pro-inflammatory cytokines such as TNF-α and INF-γ would destroy the tight junction between cells, and further long-term releasing of pro-inflammatory factors followed by the increased intestinal permeability would aggravate the PCOS (69). Other studies suggest that the increased gut mucosal permeability would promote the LPS into the systemic circulation and mediating the chronic inflammatory which is responsible for the PCOS (70).

## Potential Therapeutic Opportunity

As the increasing studies focus on the mechanism of microbiota and human diseases (71–73), the potential treatment options also have drawn great attention (74). Many studies investigated the potential effected of fecal microbiota transplantation against human disorders (75). In briefly, fecal microbiota transplantation is conducted by transplanting the microorganisms from the feces of healthy donors to a recipient’s small intestine. This treatment aims change the composition of the new host’s gut microbiome composition rapidly and treat the diseases effectively (76). Undoubtedly, fecal microbiota transplantation could represent a potential innovative therapeutic opportunity for PCOS.

Some studies reported that Lactobacillus transplantation *via* fecal microbiota transplantation could decrease the serum androgen levels and increase the estrogen levels in PCOS-induced rats (77), which form the regulated menstrual cycle and subsequently draw beneficial effect eventually in PCOS-induced rats (43). Pedro J Torres found that exposure to a healthy gut microbiome could protect against the reproductive and metabolic dysregulation in a PCOS mouse model, which suggests that healthy gut microbiome is associated with improving phenotype of PCOS (78). Wang reported that dietary α-linolenic acid-rich flaxseed oil exerts beneficial effects on PCOS through sex steroid hormones-microbiota-inflammation axis in rat model (79). And Zhang reported that probiotic bifidobacterium lactis V9 regulates the secretion of sex hormones in PCOS patients through the gut-brain axis (80). Even though the inspiring results in animal experiments, there are little clinical reports about the fecal microbiota transplantation in PCOS women. Further prospective study should be necessary in order to verify the effectiveness of the fecal microbiota transplantation on humans.

## Summary and Perspectives

As a kind of endocrine and metabolic disorder which disturbs numerous females at reproductive age, PCOS has drawn widespread attention for the reason that its relationship with menstrual cycle and fertility. In spite of the acknowledgement of PCOS as well as the development of therapeutics, some limitations including unsatisfactory cure rate, undiscovered mechanism and disorders relapse still remain.

Though an increasing studies demonstrate the correlation between the microbiota (including gut microbiota and vaginal microbiota) and PCOS, most of the studies only draw the conclusion that the diversity of microbiota changes in PCOS women. The conclusions that are widely recognized clarify the low level of α diversity, β diversity and Lactobacillus in PCOS, also the high level of Chlamydia trachomatis and Prevotella in PCOS. However, the underlying molecular mechanism of microbiota in the development of PCOS is still uncertain. In addition, most of the current studies of microbiota in PCOS still remains the level of demonstration of the relationship in human population. Even through diverse aspects (including obesity, androgen, insulin resistance, gut–brain axis and so on) were discussed in the field, only a small portion involved the mechanism research in the animal model. Thus, more molecular mechanism researches and human-related researches about the microbiota and PCOS are urgently needed. How to alleviate PCOS through regulating microbiota, how to further exploit and produce engineered microbiota are potential issues in the field. All in all, various kinds of microbiota play vital roles in regulating a variety of physiological homeostasis and pathological dysbiosis in women health through the numerous pathways, which provides us a potential insight to better understand and subsequent treat PCOS.

## Author Contributions

YG: writing-original draft. GZ: writing-original draft and editing. FZ: writing-review and editing. YL: writing-review and editing. QW: review and editing. HH: review and editing. YZ: review and editing. CM: review and editing. JD: review and editing. KH: writing-review and editing and supervision. All authors contributed to the article and approved the submitted version.

## Conflict of Interest

The authors declare that the research was conducted in the absence of any commercial or financial relationships that could be construed as a potential conflict of interest.

## Publisher’s Note

All claims expressed in this article are solely those of the authors and do not necessarily represent those of their affiliated organizations, or those of the publisher, the editors and the reviewers. Any product that may be evaluated in this article, or claim that may be made by its manufacturer, is not guaranteed or endorsed by the publisher.
